# Topography of Slow Sigma Power during Sleep is Associated with Processing Speed in Preschool Children

**DOI:** 10.3390/brainsci5040494

**Published:** 2015-11-04

**Authors:** Margaret R. Doucette, Salome Kurth, Nicolas Chevalier, Yuko Munakata, Monique K. LeBourgeois

**Affiliations:** 1 Sleep and Development Laboratory, Department of Integrative Physiology, University of Colorado Boulder, Boulder, CO 80309, USA; E-Mails: margaret.doucette@colorado.edu (M.R.D.); salome.kurth@colorado.edu (S.K.); 2 Department of Psychology, University of Edinburgh, Edinburgh EH8 9JZ, UK; E-Mail: nicolas.chevalier@ed.ac.uk; 3 Department of Psychology and Neuroscience, University of Colorado Boulder, Boulder, CO 80309, USA; E-Mail: munakata@colorado.edu

**Keywords:** early childhood, preschool children, sleep spindles, sigma power, high density EEG, processing speed, cognition

## Abstract

Cognitive development is influenced by maturational changes in processing speed, a construct reflecting the rapidity of executing cognitive operations. Although cognitive ability and processing speed are linked to spindles and sigma power in the sleep electroencephalogram (EEG), little is known about such associations in early childhood, a time of major neuronal refinement. We calculated EEG power for slow (10–13 Hz) and fast (13.25–17 Hz) sigma power from all-night high-density electroencephalography (EEG) in a cross-sectional sample of healthy preschool children (*n* = 10, 4.3 ± 1.0 years). Processing speed was assessed as simple reaction time. On average, reaction time was 1409 ± 251 ms; slow sigma power was 4.0 ± 1.5 μV^2^; and fast sigma power was 0.9 ± 0.2 μV^2^. Both slow and fast sigma power predominated over central areas. Only slow sigma power was correlated with processing speed in a large parietal electrode cluster (*p* < 0.05, *r* ranging from −0.6 to −0.8), such that greater power predicted faster reaction time. Our findings indicate regional correlates between sigma power and processing speed that are specific to early childhood and provide novel insights into the neurobiological features of the EEG that may underlie developing cognitive abilities.

## 1. Introduction

Early childhood is a period of rapid cognitive development, which facilitates increasing flexibility as children engage in more complex interactions with their environment. Cognitive development is in part determined by maturational changes in processing speed, a construct reflecting the time it takes to conduct mental operations [1,2,3]. The relationship between processing speed and working memory capacity [4,5] supports the assumption that more information is accessible before being lost through interference or decay [6], which increases the capacity to perform concurrent and more complex operations [7]. As a consequence, a faster processing speed improves the ability to reason and solve problems [6,8]. Therefore, maturational changes in processing speed have a crucial impact on cognitive performance and its development [1,5,9,10,11].

Although the link between processing speed and the development of cognitive abilities is well established, the neurobiological substrates underlying this association remain poorly understood, especially in early childhood. Several studies have reported that cognitive ability involves specific structural and functional neurobiological correlates [12,13,14], and measures derived from the sleep electroencephalogram (EEG) have proven informative in specifying associations at the local level. In adolescents and adults, visually-scored or automatically-detected sleep spindles and EEG power in the sigma band are increasingly recognized correlates of cognitive ability [15,16,17,18,19]. Sleep spindles are transient bursts of ~10–16 Hz that primarily occur during Stage 2 sleep [20,21], are characterized by a waxing and waning in amplitude and are reflective of thalamocortical oscillations [20]. Spectral power in the sigma band and visually- or automatically-detected spindles are correlates of thalamocortical activity [22] and cognitive ability. Because of divergent spatial prevalence and different functional relevance, sigma power or spindles are often divided into slow (<13 Hz) and fast frequencies (>13 Hz) [23,24,25,26,27]. The topographic representation of sigma power shows a bimodal pattern, with slow frequencies over frontal regions and fast frequencies over centroparietal regions. [9,28,29]. In general, spindles become faster from childhood to adolescence [24,29,30,31], and global maturational changes in sigma power predominate in the slow sigma frequency band [32]. The topographic representation of sigma power provides insight into these age-related changes [24,33] by showing that fast sigma power increases over centroparietal areas and that slow sigma power decreases over frontal areas across childhood and adolescence [24,32].

Published findings show maturational changes in sleep spindle frequency, sigma power and topography; however, the relationship with cognitive development is supported by only a handful of studies in older children and adolescents. For example, spindles and sigma power in children and adolescents have been linked to processing speed [34], full-scale and fluid intelligence [35] and overnight enhancement in motor task accuracy [16]. In adults, spindle and sigma power associations were reported for sleep-dependent performance improvement in various cognitive tasks, including motor memory consolidation, sequence learning and semantic memory consolidation [25,36,37,38]. In children, the frequency of spindles over central regions was negatively related to working memory and planning ability [39]. Although prior studies with children and adolescents have focused mainly on contrasting global sigma/spindle measures or single EEG derivations with cognitive ability or performance benefits [40,41,42], only a few have examined the specific topographical properties of this relationship, and to our knowledge, none have been performed in early childhood. We used high-density (HD) EEG to map the association of processing speed and sleep sigma power in a cross-sectional sample of healthy preschool children. A recent HD-EEG investigation in adolescents revealed a region-specific relationship between cognitive ability and sigma power predominating over posterior regions [35]. Thus, we hypothesized regional associations between processing speed and sigma power, with a topographical focus located over posterior areas, which are known to undergo rapid maturation in early childhood (e.g., [43]).

## 2. Experimental Section

### 2.1. Participants

Families were recruited through flyers, website advertising and personal contact at community events. Participants were healthy 2- to 5-year-old children who were excluded for a personal or immediate family history of mental disorders, chronic medical conditions, developmental disabilities, physical handicaps interfering with testing, medication use affecting sleep or alertness, delivery <38 or >42 weeks, birth weight ≥5.5 lbs., regular co-sleeping (bed-sharing), a sleep schedule varying >2 h between weekdays and weekends or traveling >3 time zones during 2 months before assessments. Of the 16 enrolled participants, 10 (4.3 ± 0.3 years; 5 females) completed all study procedures, had artifact-free data and were included for analysis. All subjects scored in the non-clinical range (40.1 ± 5.6) on the Child Behavior Checklist 1.5–5 Years Total Problems Scale [44] and on the Mullen Scales of Early Learning Composite (95.1 ± 12.5) [45]. Parents signed a consent form approved by the University of Colorado Boulder Institutional Review Board. Study procedures were performed according to the Declaration of Helsinki.

### 2.2. Procedure

Children followed an individualized, strict sleep schedule for the duration of the study, which was scheduled according to habitual bedtimes and included a maximal bedtime deviation of 20 min. This stabilization period provided minimization of sleep restriction and entrainment of the circadian system. Adherence to the sleep schedule was verified with actigraphy, parent-completed sleep diaries and daily calls/emails to the laboratory. Children wore actigraphs (AW Spectrum, Philips/Respironics, Pittsburg, PA, USA) on their non-dominant wrist for at least 5 days before sleep and processing speed assessments. Colorful Lycra bands were wrapped around the actiwatch to secure the device to children’s wrists and to prevent any skin irritation. The study protocol involved a laboratory cognitive assessment and an at-home all-night sleep EEG recording. The 30-min cognitive assessment was completed in the morning (starting at 10:00–11:00) and administered by the same researcher for all children. A parent was present during testing but was instructed to refrain from interacting with the child. Sleep was monitored during one night using HD-EEG (128 channels) in the children’s homes. The order of the two assessments was counterbalanced and separated by no more than 2 weeks. The following steps were used to minimize potential first-night effects: sleep assessments were performed in the children’s habitual sleep environment (their own bed); at-home visits during the pre-study training interval included a playful step-by-step familiarization of children with the electrode net; and families were provided with Surgilast “mock” nets, which children wore several times while sleeping before the assessments.

All sleep recordings were performed between 20:00 and 08:00 according to the children’s individual sleep stabilization schedules (see Table 1, time in bed). Sleep EEG was recorded with a portable HD-EEG system (Electric Geodesic Inc., Electrical Geodesics Sensor Net for long-term monitoring, 128 channels). Electrode nets were adjusted to the vertex and mastoids and filled with gel electrolyte. Impedances were set below 50 kΩ.

**Table 1:** Visually-scored sleep variables (mean ± SD) for whole-night data (*n* = 10).

Sleep Variables	Mean ± SD	Min	Max
Time in bed (min)	609.2 ± 67.4	483.7	647.0
Total sleep time (min)	545.3 ± 77.2	390.0	619.7
Stage 1 (%)	1.1 ± 0.3	0.7	1.6
Stage 2 (%)	35.4 ± 4.6	27.3	44.2
SWS (%)	31.0 ± 7.3	21.1	41.6
REM sleep (%)	32.5 ± 6.1	21.8	41.8
NREM sleep (%)	66.4 ± 6.1	48.2	68.3
Sleep cycle duration (min)	83.3 ± 19.3	50.5	120.4

Sleep stages are presented as a percentage of total sleep time; SWS, slow-wave sleep as non-rapid eye movement Stage N3 sleep; REM sleep, rapid eye movement sleep; NREM sleep, non-rapid eye movement sleep.

### 2.3. Measures

#### 2.3.1. Processing Speed

**Figure 1:**
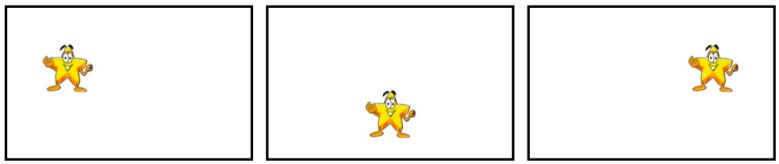
Computer task to measure simple reaction time. Children were presented with a single star stimulus that changed locations on the screen after a random time delay and were instructed to place a stylus on each stimulus that appears.

To assess processing speed via response time in preschool-age children, a task must not rely on reading or identification skills for letters or numbers (e.g., [5]). Thus, we assessed processing speed using a standard age-appropriate touch screen computer task that was designed to quantify simple reaction time [46]. We used E-Prime Version 1.2 (Psychology Software Tools, Pittsburgh, CA, USA) for stimulus presentation and response sampling. A practice trial was first administered to ensure children comprehended the task. The formal testing trial included on average a total of 13.4 ± 1.35 trials. Stimuli were presented at a viewing distance of ~60 cm (Figure 1). At the beginning of the trial, children were asked to place a stylus held in their dominant hand on a stimulus in the center of the screen. The initial stimulus disappeared upon the child placing the stylus on the image. After a random delay, a stimulus appeared in a new position, and children were instructed to lift the stylus as quickly as possible and place the stylus on the new stimulus. The time between lifting the stylus from the previous stimulus and tapping the stylus on the new stimulus was assessed as simple reaction time measured in milliseconds. Although this task has not been standardized, it has adequate convergent validity with other processing speed measures, as well as good discriminant validity with higher cognitive function tasks in a previously published study [46]).

#### 2.3.2. Processing and Analysis

Simple response times were trimmed to remove outliers (values greater than M (mean) + 3 SD) in an iterative way (*i.e.*, recalculating M and SD values after outlier removal) until no values qualified as an outlier anymore (13.3% of the values were removed). This method ensured equally strict outlier identification and removal for all participants, regardless of differences in baseline reaction times. Remaining values were averaged for each participant.

The EEG was referenced to the vertex (Cz) and sampled at 500 Hz (0.01–200 Hz). The signal was bandpass filtered (0.5–50 Hz), down-sampled to 128 Hz, and artifacts were rejected on a 20-s basis after visual inspection and if power exceeded a threshold based on a moving average in the 0.75–4.5 and 20–30 Hz bands [47]. Poor quality EEG channels were excluded (on average of 12.4 ± 9.6 channels per subject) if the channel maintained a bad signal at any time during the night. All subsequent analyses were then based on data that were re-referenced to the mean signal calculated across all channels: for all EEG samples, the signal in each channel was divided by the average across all 109 channels above the ears using our previously published methods [32].

**Figure 2:**
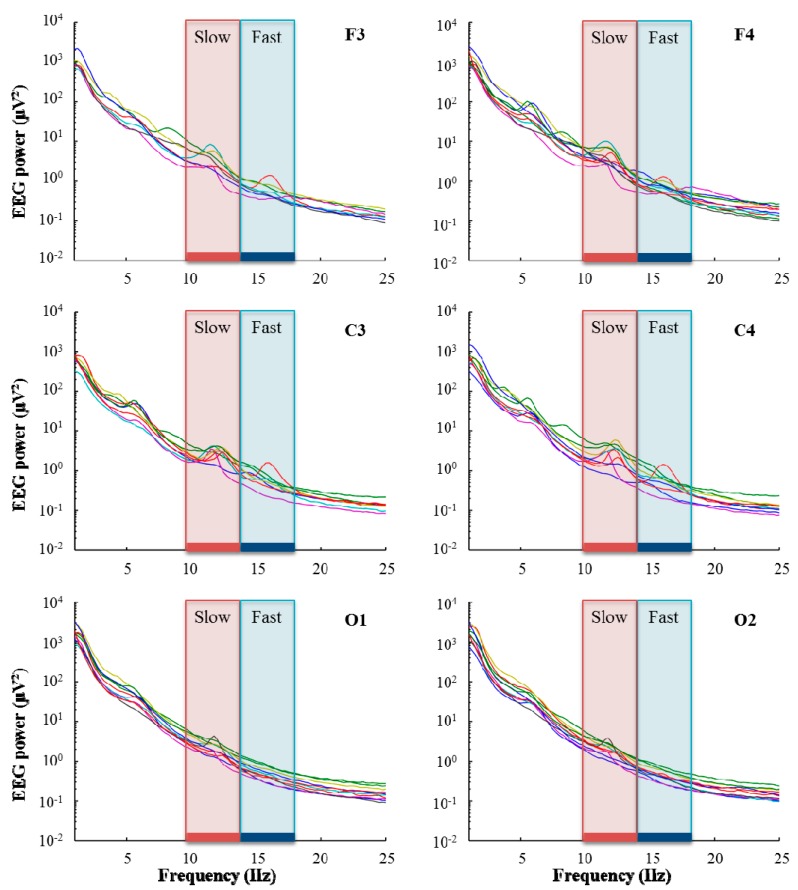
EEG power spectra during NREM sleep (*n* = 10). Electrodes F3, F4, C3, C4, O1 and O2 for the longest common duration (3.23 h) of artifact-free NREM sleep Stages 2 and 3. Based on the occurrence of individual peaks in EEG power, slow sigma frequency (red) was determined as 10–13 Hz and fast sigma frequency (blue) as 13.25–17 Hz. Data underwent artifact exclusion (see Experimental Section); channels excluded for subjects are not illustrated here.

The EEG was then visually scored for sleep stages (20-s epochs) using standard criteria [48]. Non-rapid eye movement (NREM) sleep episodes were also defined according to standard criteria [21,49]. To account for the frequently-observed “skipped” rapid eye movement (REM) sleep in children, the first NREM sleep episode was manually subdivided as in our previously published paper [32]. Specifically, a “skipped REM” was established if: (1) the duration of the first NREM episode exceeded 120 min; and (2) Stage 3 sleep in the first NREM episode was disturbed for at least 12 continuous minutes of Stage 1 sleep, Stage 2 sleep, wakefulness or movement time. If both criteria were met, the first NREM episode was subdivided at the lowest point of slow wave activity (SWA). In this sample, we identified skipped REMs in 20% of the recordings. Spectral analysis was performed for all channels (fast Fourier transform routine, Hanning window, 20-s epochs (averages of five 4-s epochs), frequency resolution of 0.25 Hz), for quantitative analysis of the EEG. The maximal common duration of artifact-free NREM sleep was included for analysis (*i.e.*, 3.23 h). We used a data-driven approach to determine the frequency ranges of slow and fast sigma bands: a visual inspection of individual EEG power spectra revealed sigma power peaks for each child (Figure 2). In order to include these individual characteristics in power spectra, we chose 10–13 Hz as the slow sigma frequency range and 13.25–17 Hz as the fast sigma frequency range.

To examine the topographical location of EEG power, we calculated maps for EEG power in the slow and fast sigma band. Statistical analysis was performed using a two-step approach similar to previous studies [50,51,52,53,54,55]. First, Pearson correlations were used to contrast reaction times with sigma power maps, and uncorrected significant effects were identified based on an alpha level of 0.05 (one-tailed tests were used because we expected a directional relationship, *i.e.*, better cognitive performance with increased sigma power [35]). Analysis of clusters within posterior regions that survived the initial threshold were then submitted to a correction at the family-wise error rate of *p* < 0.05, for the cluster level [52,53,55]. This two-step approach permitted strict assessment of our hypothesis-driven region of interest target, while still allowing an exploratory investigation of associations outside this region. All analyses were performed with the software package MATLAB (MathWorks, Version R2013a, Natick, MA, USA). Summary measures are presented as means (M) and standard deviations (SD).

## 3. Results and Discussion

**Figure 3:**
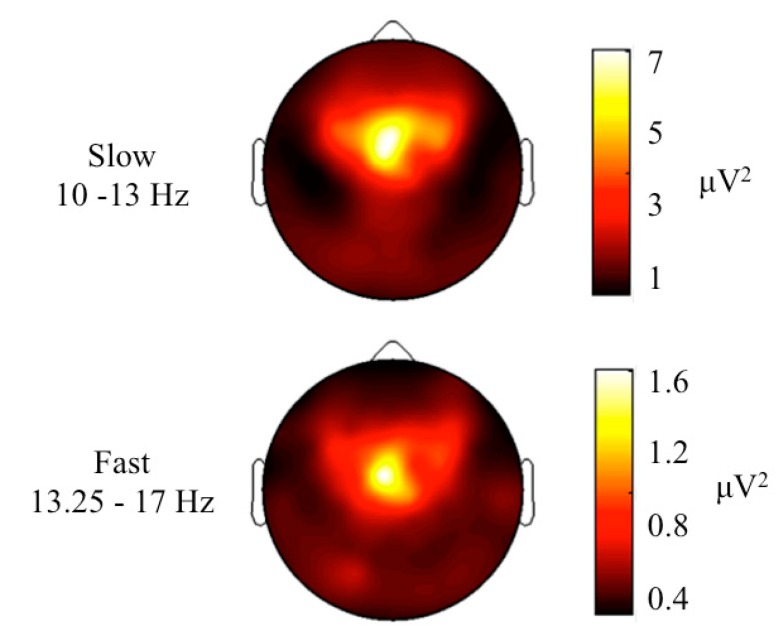
Maps of EEG power in the sigma frequency band during NREM sleep for slow (10–13 Hz) and fast sigma power (13.25–17 Hz, *n* = 10). Maps are based on 109 electrode derivations (only channels above the ears; see Experimental Section) from the maximal common duration of artifact-free NREM sleep Stages 2 and 3. Values are color coded (maxima in white, minima in dark brown) and illustrated on the planar projection of a hemispheric scalp model. Each map was proportionally scaled between minimal and maximal values, and data between the electrodes were interpolated.

**Figure 4:**
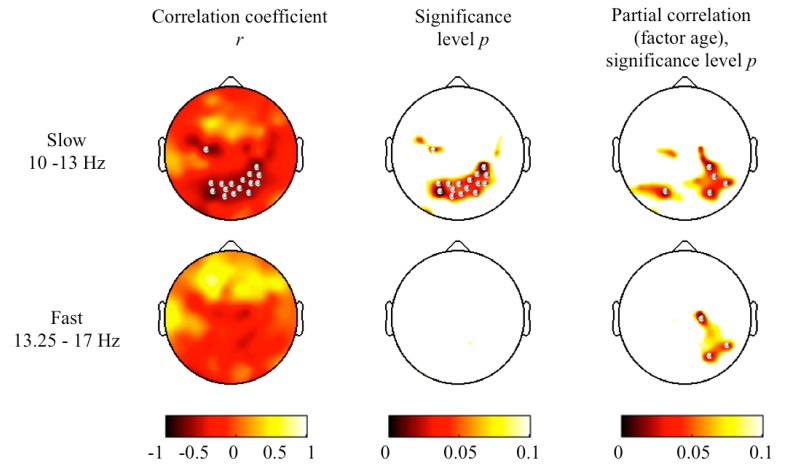
Topographical maps of Pearson correlations between processing speed and slow sigma power (10–13 Hz) and fast sigma power (13.25–17 Hz). Left column: maps for Pearson correlation coefficients (*r*); middle column: corresponding *p*-value; right column: maps for *p*-values from partial correlations (factor “age”). Power maps were individually scaled for the data range. Significant correlations (*p* < 0.05, one-tailed) are indicated with bullets.

Participants spent on average 90% ± 3.7% of their time in bed sleeping and 66% ± 6.1% of their sleep time in NREM sleep (Table 1). Slow sigma power was 4.0 ± 1.5 μV^2^, and fast sigma power was 0.9 ± 0.2 μV^2^ (average across all channels). A clear peak in the slow sigma frequency range was identified in the power spectra for all children, while for many, there was no clear peak in the fast sigma frequency range (Figure 2). In both the slow and the fast sigma frequency range, power maxima were located over central areas and were symmetrically distributed (Figure 3). The average reaction time was 1408.8 ± 251.4 ms and was only significantly correlated with slow sigma power in a cluster of 16 electrodes (*r* ranging from −0.60 to −0.80, *p* < 0.05; Figure 4), such that increased slow spindle power predicted faster processing speed (*i.e.*, shorter reaction time). In other words, 36%–64% of the variability in processing speed was explained by slow sigma power. This pattern was specific to parietal regions and was not driven by inter-individual differences in maturation, as examined by controlling for age (*p* < 0.05; partial correlations with factor “age”; Figure 4). To examine the robustness of these associations, the data were submitted to a secondary level of family-wise error correction across all 16 electrodes within the identified cluster, which revealed the stability of the association (*p* < 0.05). In the fast sigma frequency range, a relationship was found between power and processing speed when controlling for age. The topographical effects in the slow and fast sigma frequency bands were driven by NREM stage 2 sleep (N2) and largely disappeared when NREM stage 3 sleep (N3) was examined separately (Supplementary Material, Figures S1 and S2).

Although the sample size of this study (five males, five females) limits the reliability of examining sex differences, our exploratory analyses showed none with regard to processing speed. Further, the relationship between processing speed and slow sigma power remained when controlling for sex (partial correlation, factor “sex”, Supplementary Material, Figure S3). In contrast, power in the fast sigma range did not reveal a topographical relationship with processing speed when sex is included as a control variable.

This study utilized cross-sectional data from a sample of preschool children to examine the relationship between sleep sigma power and cognitive ability, as measured by processing speed. Children with faster processing speed exhibited higher slow sigma power over a parietal region, indicating a regional relationship specific to early human development. We also found a relationship between fast sigma power and processing speed when controlling for age. The distinct maturational patterns of slow and fast sigma power and their dissimilar associations with processing speed support the assumption that EEG power in the sigma frequency range reflects the functional integration of neuronal circuits underlying cognition and the maturation of these circuits.

We report a region-specific relationship between slow sigma power and cognitive ability in preschool children, which extends previous topographical observations in adolescents [35] and links between cognitive ability and central spindle frequency in older children [39]. The local effect over parietal regions in preschool children stands in contrast to predominantly frontal associations between cognition with spindles and with sigma spectral power in adults [56]. Our results imply that neural circuits underlying cognitive ability undergo associations with distinct regions across maturation and are possibly related to gray and white matter development and their functional integration [43,57]. We used an age-appropriate computer task to assess processing speed, which revealed results consistent with previous data on this age [58,59,60]. In contrast to Geiger *et al.*, who used the Wechsler Intelligence Scales for Children to measure cognitive ability via fluid intelligence, verbal intelligence, speed of processing, working memory and full-scale IQ (Intelligence Quotient) [35], we measured processing speed as simple reaction time, with the intent of assessing the most elementary component of cognitive ability. Simple reaction time was chosen because it minimizes additional cognitive demands, such as motor response execution and goal maintenance [5]. In addition, simple reaction time predicts performance on more elaborate tasks, such as higher level cognitive constructs of processing speed and working memory capacity [61].

Our findings also highlight that the region-specific association between sigma power and processing speed is driven by sleep Stage N2 and largely disappears in N3. Similarly, Nader *et al.* [34] found that slow spindle density was correlated with processing speed in Stage 2 and REM sleep, but not in slow wave sleep. They did, however, find that fast spindle density was only correlated with processing speed in slow wave sleep. In addition to using different measures (sigma power *versus* spindle density), Nader *et al.* studied adolescents, which may explain the lack of a relationship between processing speed and spindle power in slow wave sleep in our sample of preschool children.

The pacemaker of sleep spindles lies in the intrinsic properties and connectivity networks of thalamocortical neurons [62,63]. Although our data are not suited to further elucidate the development of these cellular mechanisms (reviewed in [64]), they reveal that associations between sigma power and cognitive ability occur in distinct locations in children as compared to adults. Mapping these relationships may uncover maturation processes in parieto-thalamocortical projections that are restricted to early periods of development, when the projections to frontal regions (as found in adults) are not fully mature. The parietal cortex serves an important role in integrating sensory input into motor output, which is a key process underlying performance in the reaction time task. Connectivity between parietal and prefrontal cortices may play a crucial role when executing such tasks. For example, faster processing speed has been related to increased parietal activity and decreased prefrontal activity, suggesting greater efficiency of neural interconnections between regions that mediate performance [65]. The integrity of white matter, which is linked to major cognitive developmental milestones [66], may coordinate this functional synchrony/communication and result in faster and more precise task execution.

Our findings support the claim that EEG power in the slow and fast sigma bands has distinct origins and functions [24,25,26,27]. Slower spindles were suggested to mark slower learning and the immaturity of networks involved in motor skills [16]. Though this may be the case in school-age children (10.7 ± 0.8 years), the present study examined a younger age group (4.3 ± 0.3 years). In this age group, it is likely that the fast sigma band has not fully developed yet, as suggested by cross-sectional data showing that from childhood to adolescence, spindles become faster [24,29,30,31], with an increase in fast sigma power for centro-parietal areas [24,32]. Relatedly, the differentiation of sigma power into fast and slow frequencies in the EEG power spectrum [67] and the predominance of the peak in the slow sigma frequencies suggest that slow sigma power is a strong indicator of early individual differences in cognitive ability that is not driven by inter-individual differences in maturation, as examined by controlling for age. In contrast, the relationship between fast sigma power and cognitive ability is intertwined with age-related changes, as shown by correlations that were only evident when variance was accounted for by age.

Mapping of slow sigma power elucidates anatomical and functional properties of the thalamocortical system related to cognitive abilities. The relationship between thalamocortical network activity and cognition, memory and attention is well established [68,69,70,71], and slow sleep spindles in particular are linked to thalamocortical efficiency [18] and to higher cognitive abilities [72]. Finally, characterizing target network coordination through mapping sigma power across development may provide a normative benchmark for understanding atypical neurodevelopment. For example, adults with schizophrenia reveal strong correlations between mediodorsal thalamic volume and frontal spindle activity [22].

This study used objective methods to investigate the relationship between region-specific brain activity during sleep and cognitive performance in a largely understudied age group. Because collecting sleep HD-EEG data in preschool children is challenging and published data are scarce, this unique database is a rich source for preliminary insights into sleep and cognitive ability in the early years of life. We were also able to obtain high sleep quality in our sample, as confirmed by sleep architecture measures similar to earlier reports in preschool children [26,73]. Nonetheless, these findings should be interpreted in the context of a limited sample size. To reduce the likelihood of a type I error, we applied a two-step statistical approach that permitted a strict assessment of our hypothesis. Additionally, we included spectral EEG activity between 10 and 17 Hz, which does not distinguish between background EEG activity and phasic spindle activity. Further, because behavioral testing did not directly precede the sleep recording, interference during the time between assessments could have occurred. Finally, these data are correlative and, thus, cannot infer causality between sigma power and processing speed. Thus, further research is needed to elucidate neuronal correlates for the different and frequently-used sleep spindles measures, their detection algorithms and calculations of sigma power.

## 4. Conclusions

Our findings in healthy preschool children demonstrate that both slow and fast sigma power predominate over central areas and that parietal slow sigma power predicts 36%–64% of the variability in processing speed. These data extend previous research by considering regional associations between sigma power and cognitive performance in the understudied preschool years. Furthermore, slow sigma power may be a neuronal characteristic closely related to cognitive development in early childhood that has the potential to provide a normative benchmark for understanding atypical brain network maturation.

## Supplementary Files

Supplementary File 1
