# Supplementary material for: Topography of Slow Sigma Power during Sleep is Associated with Processing Speed in Preschool Children
Source: Brain Sci. 2015 Nov 4;5(4):494–508. doi: 10.3390/brainsci5040494 (PMC4701024; doi:10.3390/brainsci5040494)
Supplement: Supplementary File 1 [file brainsci-05-00494-s001.doc]

Supplementary Materials


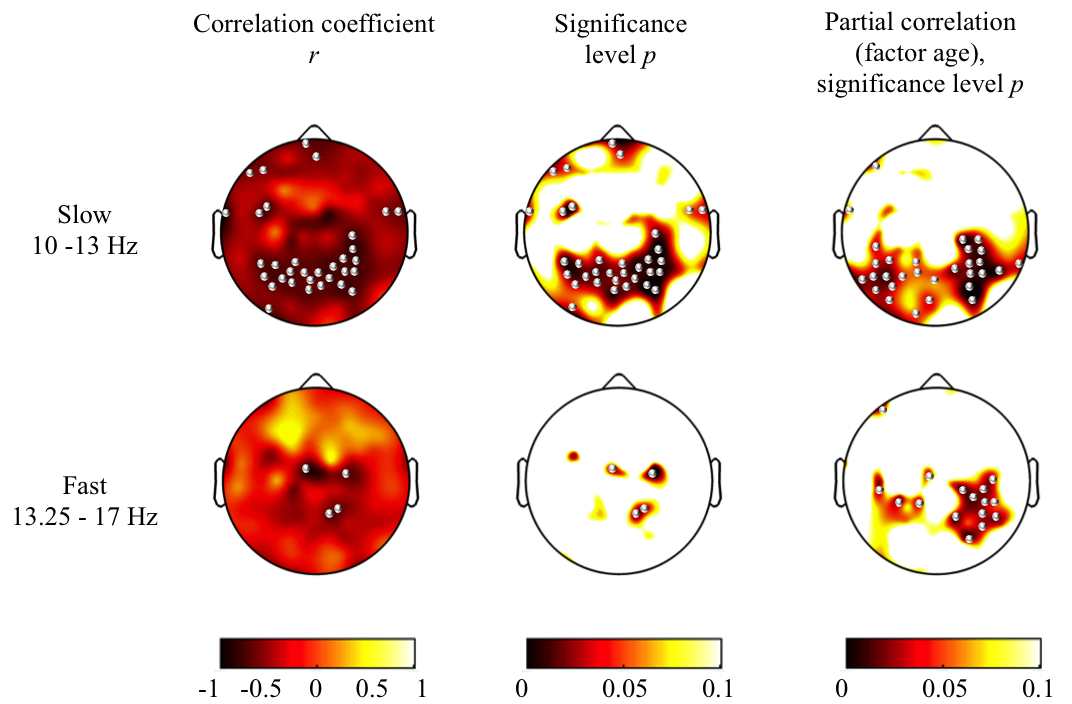


**Figure S1.** Topographical maps of Pearson correlations between processing speed and slow sigma power (10–13 Hz) and fast sigma power (13.25–17 Hz) for sleep stage N2. **Left column**: Maps for Pearson correlation coefficients (*r*); **Middle column**: Corresponding *p*-value; **Right column**: Maps for *p*-values from partial correlations (factor “age”). Power maps were individually scaled for data range. Significant correlations (*p* < 0.05, one-tailed) are indicated with bullets. Correlations between processing speed and slow sigma power were found in 34 electrodes (*r* ranging from −0.6 to −0.9) and in 30 electrodes after controlling for age (*r* ranging from −0.6 to −0.9). Fast sigma power was correlated with processing speed in 4 electrodees (*r* ranging from −0.7 to −0.8) and in 16 elecrodes after controlling for age (*r* ranging from −0.6 to −0.9).


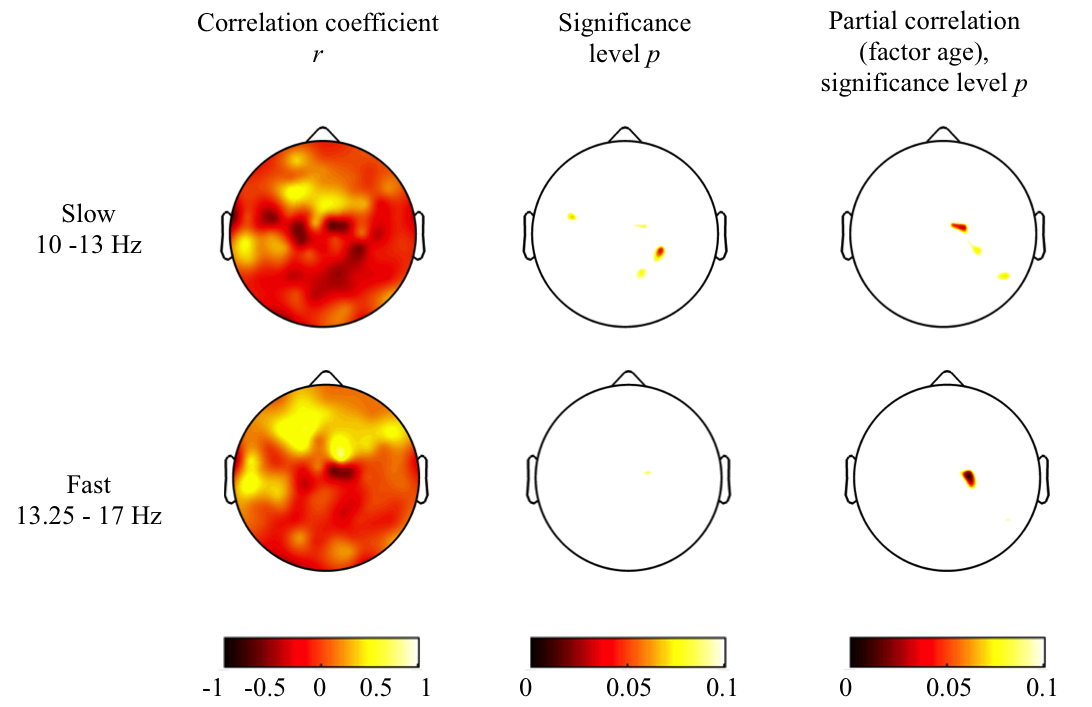


**Figure S2.** Topographical maps of Pearson correlations between processing speed and slow sigma power (10–13 Hz) and fast sigma power (13.25–17 Hz) for sleep stage N3. **Left column**: Maps for Pearson correlation coefficients (*r*); **Middle column**: Corresponding *p*-value; **Right column**: Maps for *p*-values from partial correlations (factor “age”). Power maps were individually scaled for data range. No significant correlations (*p* < 0.05,
one-tailed) were found.


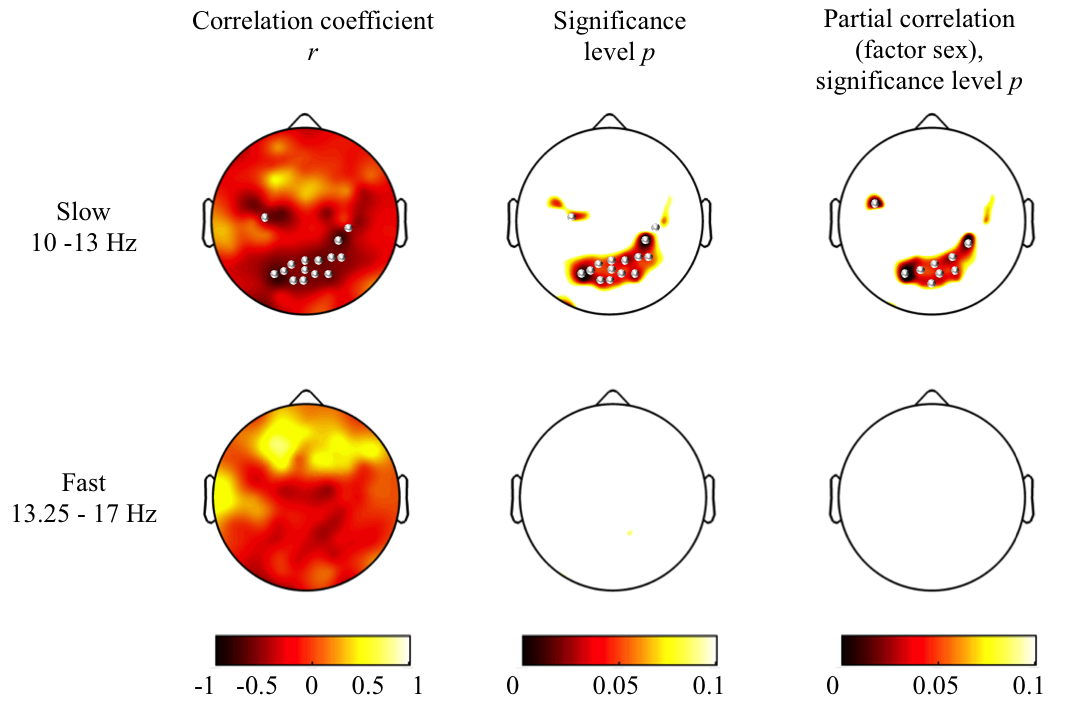


**Figure S3.** Topographical maps of Pearson correlations between processing speed and slow sigma power (10–13 Hz) and fast sigma power (13.25–17 Hz) for NREM sleep
(N2 and N3). **Left column**: Maps for Pearson correlation coefficients (*r*); **Middle column**: Corresponding *p*-value; **Right column**: Maps for *p*-values from partial correlations (factor “sex”). Power maps were individually scaled for data range. Significant correlations
(*p* < 0.05, one-tailed) are indicated with bullets. Slow sigma power and processing speed were correlated in 15 electrodes (*r* ranging from −0.6 to −0.8) and in 9 electrodes after controlling for age (*r* ranging from −0.6 to −0.9).
